# Mitochondrial Dysfunction in Vascular Wall Cells and Its Role in Atherosclerosis

**DOI:** 10.3390/ijms22168990

**Published:** 2021-08-20

**Authors:** Diana Salnikova, Varvara Orekhova, Andrey Grechko, Antonina Starodubova, Evgeny Bezsonov, Tatyana Popkova, Alexander Orekhov

**Affiliations:** 1 Faculty of Medicine, Lomonosov Moscow State University, 119192 Moscow, Russia; dianasalnikova08@yandex.ru; 2 Laboratory of Oncoproteomics, Institute of Carconigenesis, N. N. Blokhin Cancer Research Centre, 115478 Moscow, Russia; 3 Laboratory of Angiopathology, Institute of General Pathology and Pathophysiology, 125315 Moscow, Russia; evgeny.bezsonov@gmail.com (E.B.); a.h.opexob@gmail.com (A.O.); 4 Federal Scientific Clinical Center for Resuscitation and Rehabilitation, 109240 Moscow, Russia; noo@fnkcrr.ru; 5 Federal Research Centre for Nutrition, Biotechnology and Food Safety, 109240 Moscow, Russia; avs.ion@yandex.ru; 6 Therapy Faculty, Pirogov Russian National Research Medical University, 117997 Moscow, Russia; 7 Institute of Human Morphology, 117418 Moscow, Russia; 8 V. A. Nasonova Institute of Rheumatology, 115522 Moscow, Russia; popkovatv@mail.ru

**Keywords:** mitochondrion, mitochondrial DNA, oxidative stress, inflammation, atherosclerosis

## Abstract

Altered mitochondrial function is currently recognized as an important factor in atherosclerosis initiation and progression. Mitochondrial dysfunction can be caused by mitochondrial DNA (mtDNA) mutations, which can be inherited or spontaneously acquired in various organs and tissues, having more or less profound effects depending on the tissue energy status. Arterial wall cells are among the most vulnerable to mitochondrial dysfunction due to their barrier and metabolic functions. In atherosclerosis, mitochondria cause alteration of cellular metabolism and respiration and are known to produce excessive amounts of reactive oxygen species (ROS) resulting in oxidative stress. These processes are involved in vascular disease and chronic inflammation associated with atherosclerosis. Currently, the list of known mtDNA mutations associated with human pathologies is growing, and many of the identified mtDNA variants are being tested as disease markers. Alleviation of oxidative stress and inflammation appears to be promising for atherosclerosis treatment. In this review, we discuss the role of mitochondrial dysfunction in atherosclerosis development, focusing on the key cell types of the arterial wall involved in the pathological processes. Accumulation of mtDNA mutations in isolated arterial wall cells, such as endothelial cells, may contribute to the development of local inflammatory process that helps explaining the focal distribution of atherosclerotic plaques on the arterial wall surface. We also discuss antioxidant and anti-inflammatory approaches that can potentially reduce the impact of mitochondrial dysfunction.

## 1. Introduction

Atherosclerosis with related cardiovascular diseases is still the leading cause of mortality worldwide. Prevalence of atherosclerosis increases with population ageing. Among the dangerous and life-threatening consequences of atherosclerosis are ischemic heart disease and stroke that can develop when the disease affects the arteries feeding the heart and the brain correspondingly [1]. Atherosclerosis is a chronic disease, which can remain asymptomatic during a long period of time. With the development of non-invasive diagnostic methods and accumulation of knowledge from post-mortem studies, it became clear that the disease begins at a much younger age than previously thought and can be present in young people without clinical manifestation [2,3]. Therefore, better understanding of the disease pathogenesis at the early stages is crucial for the development of effective therapies. Pathogenesis of atherosclerosis includes many known factors, such as alterations of lipid metabolism, oxidative stress, changes in the immune system functioning, and decreased tissue response to injury. Accumulating evidence reveals that atherosclerosis shares some pathophysiological mechanisms with other chronic human diseases, including cancers and neurological disorders [1,4,5].

Most of the existing therapeutic approaches are limited to alleviating atherosclerosis symptoms and slowing down their progression but offer little help against plaque development initiation. Despite the advances in our understanding of atherosclerosis mechanisms, several important questions remain unanswered. One of them is the origin of local, or even focal distribution of atherosclerotic lesions in the arterial wall [6]. Macroscopic examination of post-mortem aortic wall specimens allows distinguishing areas, unaffected by atherosclerosis or developing diffuse intimal thickening, and areas containing atherosclerotic plaques at different development stages. Microscopic study of such samples demonstrated that atherosclerotic plaque development may possibly be explained by the course of local inflammatory response. In some areas, tissue repair occurs rapidly after the inflammation is resolved, leading to diffuse intimal thickening. However, in atherosclerosis-prone areas, the inflammatory response can become chronic, which results in the formation of atherosclerotic lesions [6,7]. One of the possible explanations of such irregularity of local immune response is presence of cells affected by mitochondrial dysfunction that have altered metabolism and are subject to oxidative stress.

Mitochondria are semi-autonomous organelles that have evolved as a result of endosymbiosis. This organelle plays a key role in cell survival and death, because serves as a main source of cellular energy through respiration. Moreover, mitochondria participate in regulating cellular metabolism and ion balance and play important signaling functions. The mitochondrion has its own DNA (mtDNA) in a form of circular chromosome, which encodes the majority of (but not all) proteins necessary for respiratory chain functioning [8]. Besides energy production, mitochondrial respiratory chain also generates significant amounts of free radicals and reactive oxygen species (ROS) as by-products, serving as an important intracellular source of these reactive agents. Levels of mitochondrial ROS depends on the respiratory activity, metabolism level, and correct functioning of the mitochondria [9]. While low levels of ROS have important signaling functions, elevated ROS can have damaging effect on the surrounding cellular structures, altering DNA, proteins, and other molecules. The mtDNA, which is located close to the mitochondrial membranes where the reactions take place, is exposed to elevated ROS levels. Although questionable, such exposure is generally believed to be one of the possible explanations of increased mutagenesis rate in the mtDNA as compared to genomic DNA. Other explanations include the higher rate of errors made by mtDNA replication machinery and less reliable DNA repair system than that of genomic DNA. MtDNA mutations are can be homoplasmic or heteroplasmic depending on whether the mutated gene is present in all mtDNA copies within a cell or only in part of them. Deleterious mtDNA mutations can result in respiratory chain dysfunction, with reduced energy and increased ROS production. Moreover, deletions in the mtDNA can trigger its compensatory overproliferation (increase of copy number). Together, these processes may lead to further increase of ROS, forming a vicious circle, which can lead to cell death and damage of surrounding tissue, thus creating local pro-inflammatory conditions.

Certain mtDNA mutations can cause severe mitochondrial diseases (mitochondriopathies) that are inherited, while others can accumulate and contribute to disease development during lifetime. As mitochondria are inherited through the maternal line, so do known inherited mitochondrial diseases, such as MIDD (maternally inherited diabetes and deafness) or MELAS (mitochondrial encephalopathy, lactic acidosis, and stroke-like episodes) [10]. Although these conditions are associated with increased risk of stroke and cardiovascular events, their link to atherosclerosis remains elusive. Because mitochondrial inheritance is asexual and not accompanied by genetic recombination, it is subject to “Muller’s ratchet” effect, the irreversible accumulation of mutations [11]. This effect is likely to be more pronounced for mtDNA than genomic DNA which undergoes recombination. However, genetic bottlenecks for mtDNA during mammalian embryonic development have been described that consist of replication of a certain mtDNA subpopulation or reduction of copy number [11]. Mutagenesis in mtDNA and accumulation of mtDNA mutations occurs during lifetime in different parts of the body, and this process can be enhanced by external factors, such as smoking or infections [12].

The list of mtDNA mutations involved in human diseases is growing, despite the methodological challenges of mitochondrial mutation research. Since mitochondrial dysfunction can be compensated at different levels, functional analysis of mtDNA mutations requires creating cellular and animal models that allow demonstrating the effect of the studied mutation. In particular, cytoplasmic hybrids (cybrids) are powerful modern tools that are used to classify and study mtDNA mutations. Cybrid models make it possible to investigate functional significance of mtDNA mutations in a given cell type [13].

Therefore, mitochondria can be regarded as disease-modifying agents in chronic disorders. Mitochondrial dysfunction is one of the reasons for phenotypic differences of individuals, tissues, and cells. Restoration of mitochondrial function using different strategies is considered as attractive therapeutic approach, and such strategies are being actively developed [14].

## 2. Risk Factors of Atherosclerosis: Oxidative Stress and LDL Modification

Oxidative stress is an imbalance between the generation of ROS or reactive nitrogen species (RNS) and the ability of the organism to neutralize them with endogenous antioxidant systems. ROS include superoxide (O_2_•), hydrogen peroxide (H_2_O_2_), and hydroxyl radical (•OH), while RNS-nitroxyl anion (NO−), nitrosonium cation (NO+), nitrate (NO_3_−) and S-nitrosothiols (RSNO). Both ROS and RNS play important physiological roles, being involved in cell signaling, modulation of transcriptional factors, and apoptosis. Mitochondrial production of ROS is therefore tightly controlled under normal conditions. Weakening of such control, increased production of ROS/RNS or inadequate functioning of antioxidant systems lead to mitochondrial oxidative stress [9]. Increased oxidative stress was shown to be associated with various human diseases, including cancers, neurodegenerative diseases, chronic inflammation, and cardiovascular disorders. It was also shown to be a significant element in atherosclerosis pathogenesis. Moreover, major known risk factors of atherosclerosis, including dyslipidemia, diabetes, and hypertension are all associated with increased production of ROS [15]. Many various enzymes are involved in oxidative stress development in the vascular system, for example, NADPH oxidase, xanthine oxidase, and an endothelial NO synthase. However, the mitochondrial respiratory chain enzymes are among the most potent producers. Mitochondrial oxidative stress can exacerbate mitochondrial dysfunction by damaging mtDNA and organelle structures therefore forming a vicious cycle [14,16]. In atherosclerosis, deficiency of antioxidant systems can promote disease progression through oxidative stress. For instance, these processes were observed in atherosclerosis models, such as *apoe ^−/−^* atherosclerotic mice deficient for superoxide dismutase-2 (SOD-2) [17,18].

**Figure 1:**
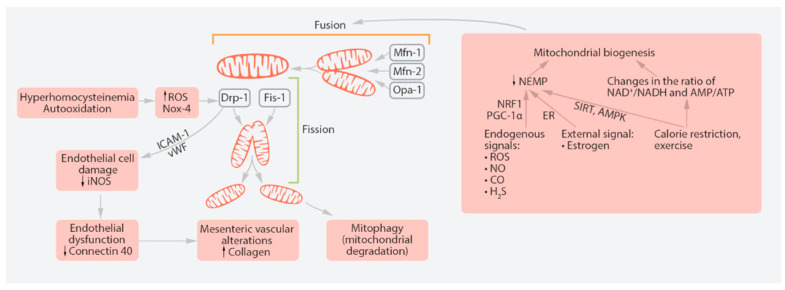
Mitochondrial fission and fusion and their place in cellular processes. Mitochondria constantly undergo fission and fusion in a dynamic process. Fission is mediated by Drp-1 (dynamin-related protein 1) and Fis-1 (mitochondrial fission 1 protein). The key proteins of fusion are Mfn-1 (mitofusin-1), Mfn-2 (mitofusin-2) and Opa-1 (Optic Atrophy 1). Hyperhomocysteinemia is a risk factor for peripheral vascular disorders. Endothelial dysfunction-related proteins are iNOS (inducible nitric oxide synthase), ICAM-1 (inter-cellular adhesion molecule 1) and vWF (von Willebrand factor). Nox-4 is a gene that encodes NADPH-oxidase 4, which, in turn, produces reactive oxygen species (ROS). AMPK, AMP-activated protein kinase; ER, estrogen receptor; NEMP, nuclear-encoded mitochondrial protein; NRF1, nuclear respiration factor-1; PGC-1α, peroxisome proliferator-activated receptor gamma coactivator 1-alpha.

Under normal conditions, mitochondrial antioxidant and repair systems are able to counterbalance the deleterious effects of excessive ROS. Moreover, if part of mtDNA pool and organelle structures are damaged, they can be recycled through the process of mitochondrial turnover. Within the cell, mitochondria undergo cycles of fission and fusion, known as mitochondrial turnover, which maintains a pool of functional organelles that corresponds to the cell’s energy needs. Excessive or damaged parts of the mitochondria are fragmented to smaller structures that can later be recycled through mitophagy, a specialized type of autophagy. These processes are executed by a set of specialized proteins, which mutations are known to block the mitochondrial turnover and cause mitochondrial disorders (Figure 1) [19]. The existing balance between fission and fusion is necessary for effective mitochondrial turnover inside the cell, maintaining a functional population of these organelles that corresponds to energy expenditure [20]. Fusion of the mitochondrial inner membrane is regulated by a dynamin protein Opa1 (Optic Atrophy 1) [21]. In cells with relatively low energy needs, mitochondrial size is usually smaller than in cells characterized by high energy consumption and active metabolism.

Disturbance of mitophagy results in accumulation of damaged and dysfunctional organelles that can act as ROS producers and can be dangerous to the cell [22]. Mitophagy is initiated through activation of P-TEN-induced kinase (PINK1), which is located at the external mitochondrial membrane, and a ubiquitin ligase PARKIN [23]. These proteins can respond to decreased mitochondrial membrane potential.

Suppression of autophagy in macrophages was shown to promote atherosclerosis development in mice that were kept on a high-fat diet or in low-density lipoprotein-deficient animals (*ldlr*^−/−^ mice). Inhibition of autophagy in these models was shown to be promoted by local inflammation, decreased efferocytosis, and reduced autophagic competence [24]. Experiments on murine macrophages have demonstrated that stimulation of mitophagy inhibited pyroptosis induced by oxidized LDL [25]. Therefore, accumulating evidence indicates that mitochondrial turnover and mitophagy are important protective mechanisms that may reduce the risk of mitochondrial dysfunction and associated disorders [26]. The role of mitophagy in the correct functioning of the innate immune system is currently widely recognized [27]. Future studies should identify and characterize the factors regulating mitochondrial turnover and the pathologic features associated with its dysfunction. Correction of these features may provide interesting possibilities for treatment of the disease at early stages.

Mitochondrial metabolic activity can change in response to cellular energy demands and external signals. However, such plasticity can also contribute to pathology development, as was shown for carcinogenesis, often associated with mitochondrial metabolic switch to glycolysis even while oxygen is present (Warburg effect) [28]. More recent studies demonstrated a crucial role of pentose phosphate pathway (PPP) in this effect, since pharmacological or genetic inhibition of this pathway reduced proliferation of several cancer lines [29]. A tight link was demonstrated between PPP activity, ROS generation, and autophagy, that may help explaining the mechanisms of cancer cells proliferation. In response to ROS elevation, cancer cells can turn from glycolysis to PPP; however, sustained oxidative stress can inhibit this pathway through glucose-6-phosphate dehydrogenase inhibition and increase autophagy [30]. The existence of similar link in atherosclerosis development remains to be demonstrated.

Besides the energy production, mitochondria also serve as important controllers of the cell fate, hence the choice between cell survival and death. Triggering of apoptosis through the mitochondrial pathway includes permeabilization of the mitochondrial outer membrane through damage or opening of the mitochondrial permeability transition (PT) pore. That leads to dissipation of the proton gradient across the membrane and uncoupling of oxidative phosphorylation, disrupting the energy production. Furthermore, water can enter the mitochondrial matrix through the open PT pore, which results in swelling of the intermembrane space, outer membrane rupture, and release of apoptosis-triggering factors into the cytoplasm. Among such factors are cytochrome c, apoptosis-inducing factor (AIF), and endonuclease G that initiate the apoptotic cascade through caspase activation [31]. Under normal conditions, apoptotic cells are cleared from the tissue by phagocytic cells, such as macrophages, which prevent inflammation and tissue damage. Phospholipid phosphatidylserine (PS) on the outer leaflet of the plasma membrane serves as a marker of apoptotic cells, which is also widely used in in vitro experiments.

The integrity of the mitochondrial membranes is also necessary for their proper functioning as important regulators of Ca^2+^ homeostasis within the cell. Mitochondria serve as cellular Ca^2+^ reservoir alongside the endoplasmic reticulum (ER), with which they form special type of contacts (mitochondrial-associated ER membranes, of MAMs). Thus, they actively participate in intracellular Ca^2+^ signaling, which regulates multiple cellular processes, including autophagy. This aspect of mitochondrial functioning appears to be interesting to study in the context of atherosclerosis, since Ca^2+^ homeostasis disturbance is important for plaque calcification and formation of unstable lesions. The role of mitochondrial Ca^2+^ homeostasis has been described in several reviews [32,33].

The involvement of oxidative stress in atherogenesis is highlighted by the important role that oxidized low-density lipoprotein (oxLDL) play in this process. Circulating LDL particles are exposed to various physical and chemical alterations in the blood flow. These changes can affect the lipid, protein and carbohydrate moieties of the lipoprotein particle. The earliest process in this modification chain appears to be desialylation, the removal of the terminal sialic acid residues from glycan chains [34]. Desialylation is followed by other modifications, accompanied by particle size reduction, density increase and acquisition of electric charge. The resulting small dense LDL (sdLDL) is characterized by reduced content of antioxidants, and is therefore subject to oxidation [35]. Moreover, modified LDL particles have prolonged residence time in the arterial wall, where oxidation is facilitated, because of their interaction with tissue components, such as glycans. Atherogenic LDL accumulates in sites of the arterial wall, which are prone to atherosclerosis. Inflammatory reaction initiated at such sites can be at least partially explained by stimulation of LDL particle phagocytosis with subsequent cytokine release and the recruitment of inflammatory cells [36]. Modified LDL can also induce endothelial dysfunction, which is considered as an early marker of atherosclerosis, as demonstrated in the J774A.1 murine macrophage cell line. MtDNA-depleted (rho0) cells appeared to be resistant to caspase-1-dependent cell death associated with DAMPs (damage-associated molecular patterns) release [37]. Further studies should investigate the effects of modified LDL uptake on mitochondrial function.

Intracellular lipid accumulation in the arterial wall occurs in different cell types. Both recruited (such as macrophages) and resident (such as modified smooth muscle cells) arterial wall cells are able to internalize modified LDL and transform to lipid-rich foam cells. Moreover, foam cells continue to produce pro-inflammatory mediators, supporting the inflammatory response. These processes lead to neointima formation through hyperplasia, migration, and proliferation of pericytes and vascular smooth muscle cells [38]. By contrast, high-density lipoprotein (HDL), apoA-I, and endogenous apoE are protective against inflammation and oxidative stress. Moreover, these molecules also promote cholesterol efflux, which helps slowing down lipid accumulation and therefore atherosclerotic lesion formation [39]. More broadly, apolipoprotein balance can be regarded as a potent regulator of the immune system. Mice lacking *apoe* and *ldlr* are widely used atherosclerosis models. It was shown that presence of *apoe* (*apoe^h/h^*, *ldlr^−/−^*) resulted in reduced plaque formation as compared to (*apoe^−/−^*, *ldlr^−/−^*) animals, with markedly reduced numbers of circulating leukocytes and pro-inflammatory monocytes. This was associated with reduced expression of pro-inflammatory molecules by both immune and endothelial cells [40]. Later studies consolidated our knowledge of apolipoproteins as important immune system modulators [41].

## 3. Mitochondrial Dysfunction and Inflammation

Accumulating evidence positions mitochondria as a key player in the development of the inflammatory response and in the maintenance of chronic inflammation. Numerous human pathologies that are associated with mitochondrial dysfunction, including cancers, neurodegenerative diseases, and atherosclerosis are also characterized by chronic inflammation. Atherosclerosis, in particular, is currently regarded as chronic inflammatory disorder, and the mechanisms of the inflammatory component of the disease are being actively studied. Modified LDL can trigger the production of autoantibodies followed by formation of circulating LDL-containing immune complexes that are highly atherogenic and pro-inflammatory [36]. Furthermore, it was shown that presence of modified LDL (such as oxLDL) stimulates phagocytosis in macrophages, therefore promoting their pro-inflammatory activation [42]. Such engulfment of large quantities of lipids leads to formation of cholesterol crystals in lipid-accumulating cells, which is another potent mechanism of inflammatory activation. In growing atherosclerotic plaques, pro-inflammatory environment is maintained by elevated production of various cytokines and chemokines by activated cells, which had been reviewed elsewhere [42].

The role of the mitochondria in inflammation became a subject of numerous reviews [43,44]. Moreover, age-associated decline of the mitochondrial function linked to the progression of chronic age-associated inflammatory disorders are now embraced in the concept of “inflammaging” that may improve our understanding and help develop refined treatment approaches, such as targeting mitochondrial oxidative stress [44]. One of the well-known pro-inflammatory features of mitochondrial dysfunction is increased mitochondrial ROS generation and oxidative stress. Moreover, mitochondria are responsible for metabolic regulation of the immune cells, including macrophages, through Krebs cycle modulation [42]. Mitochondrial damage can lead to the release of mtDNA, which is a potent DAMP recognized by the immune cells that can trigger the inflammatory response [43]. In response to DAMP, the assembly of NLRP3 inflammasome is triggered, which results in potent downstream pro-inflammatory signaling [45]. Later studies have demonstrated that mitochondrial damage can promote the NLRP3 activation directly, through the release of mtDNA, potassium ions efflux, and ROS generation [46] and also serving as a special site of inflammasome assembly [47]. However, the role of mitochondria in the immune system regulation is much more complex than mere pro-inflammatory signaling. Recent studies have demonstrated the involvement of the mitochondria in regulation of immune system homeostasis and tissue regeneration through distinct signaling pathways, such as Wnt pathway and TGFβ signaling, as reviewed elsewhere [48]. The above listed pathways can be more or less active depending on the environment and the tissue type and are being especially actively studied in cancer, although can be relevant for other human pathologies. Peroxisome proliferator activated receptor γ coactivator 1 (PGC-1) family proteins that are involved in the regulation of many cellular metabolic pathways were also shown to control mitochondrial metabolism. PGC-1α has been identified as a master regulator of mitochondrial biogenesis through transcriptional activation of nuclear respiratory factor (NRF) 1/2. This signaling pathway leads to activation of mitochondrial biogenesis through the mitochondrial transcription factor A (TFAM), which binds mtDNA, regulating its stability, replication, and transcription. The prominent role of PGC-1 proteins in the regulation of immune system is currently well known and was shown to be largely dependent on mitochondrial regulation. For instance, in macrophages, PGC-1α promotes alternative activation, thus alleviating the inflammatory response, through positive regulation of antioxidant genes and ROS reduction, while PGC-1β overexpression promotes mitochondrial biogenesis and reduces the proinflammatory cytokine generation [49].

Correction of mitochondrial dysfunction was shown to have protective anti-inflammatory effects. Signaling pathways regulating mitochondrial function are currently being investigated as potential points of therapeutic intervention for treatment of various diseases, including atherosclerosis. For instance, upregulation of PGC-1α was shown to be protective against atherosclerotic lesion development [50]. Mitophagy was found to have a potent effect inhibiting NLRP3 inflammasome activation, with potential therapeutic significance [51]. In vascular smooth muscle cells (VSMCs), mitophagy was shown to protect from apoptosis induced by pro-atherogenic modified LDL [52] or autophagy deficiency [53]. Such protective effect is likely to alleviate the inflammatory condition in atherosclerotic plaque.

**Figure 2:**
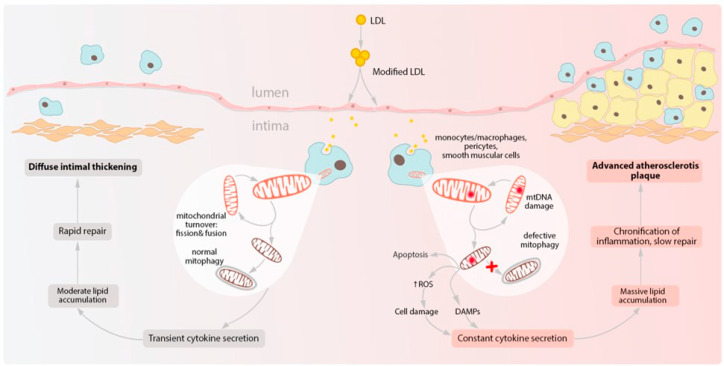
Schematic representation of processes linking mtDNA mutations with chronic inflammation. Multiply modified LDL particles being accumulated and then internalized by macrophages can change mitochondrial function which finally contributes to the formation of atherosclerotic plaques. DAMPs, damage-associated molecular patterns; LDL, low-density lipoprotein; ROS, reactive oxygen species.

In summary, mitochondrial dysfunction appears to be a key player in pro-inflammatory response and chronic inflammation in the arterial wall, which ultimately leads to atherosclerosis progression. Presence of distinct cells with dysfunctional mitochondria within the endothelial layer of the arteries may provide for an explanation of the focal distribution of atherosclerotic lesions observed on ex-vivo samples [54]. Exposure to circulating atherogenic LDL leads to lipid uptake by phagocytic cells of the arterial wall triggering an inflammatory response. However, in case of the intact mitochondrial function, this inflammation can be effectively resolved, followed by tissue repair and the formation of so called diffuse intimal thickening. By contrast, mitochondrial dysfunction in such cells (caused by mtDNA mutations, ROS elevation, and associated damage or deficient mitochondrial turnover) leads to continuous release of pro-inflammatory factors and perpetuation of the inflammatory process. In the sites where such cells are present, the inflammatory environment persists, tissue repair is deficient, and advanced atherosclerotic plaque is more likely to develop (Figure 2).

## 4. mtDNA Mutations in Atherosclerosis and Related Pathologies

In many cases, mitochondrial dysfunction is caused by mtDNA mutations. Since mtDNA is present in several copies within the cell, mtDNA mutations can be homo- or heteroplasmic, and the latter are characterized by certain heteroplasmy level, at which phenotypic effects of the mutation become apparent. Both spontaneously acquired and inherited mtDNA mutations can contribute to human disease development, with MIDD and MELAS being examples of maternally inherited mitochondrial diseases [10,55]. The list of mtDNA variants implicated in human disease is constantly growing. Mitochondrial genome contains only 37 genes, but some of them encode the key enzyme complexes required for oxidative phosphorylation: complexes I, III, IV, and V. To explain why these vital genes have not been copied to the host cell genome, the CoRR (Colocation for redox regulation of gene expression) hypothesis was proposed, according to which, such location was important for direct regulation of oxidative phosphorylation enzymes in response to changes of the redox state [56]. Apart from protein-coding genes, areas of mtDNA that are required for its replication (D-loop) and mitochondrial tRNA genes can be affected by mutagenesis with deleterious effects.

Study of mtDNA mutations accumulating in different sections of the arterial wall samples obtained from deceased patients revealed a list of mtDNA variants associated with atherosclerosis (hence found in the areas affected by atherosclerotic lesions). Among them were m.3256C>T (tRNA-Leu1 gene) and m.12315G>A (tRNA-Leu gene) encoding for tRNAs, m.3336T>C (ND1 gene), m.5178C>A (ND2 gene), m.14459G>A (ND6), and m.13513G>A (ND5), all encoding for NADPH dehydrogenase subunits, and m.15059>A in the cytochrome B gene [6]. Another study revealed that m.15927G>A mutation that affects mitochondrial threonine tRNA was associated with coronary heart disease [57]. Numerous studies identified mtDNA mutations that accumulate with age, and some of them can possibly contribute to age-associated atherosclerosis development [58,59,60].

Since multiple components of the respiratory chain are encoded by the mitochondrial genome, abnormalities of oxidative phosphorylation and excessive generation of ROS are among the frequent consequences of mtDNA mutations. Experiments with uncoupling of oxidative phosphorylation in vitro demonstrated that mutations m.del1562G, m.12315G>A, m.3256C>T, m.14459G>A, and m.13513G>A promoted proton leakage and oxygen consumption. As a result, ROS were produced in excessive amounts exacerbating mitochondrial dysfunction [14]. It was shown that m.14459G>A was associated with increased basal rate of oxygen consumption, while m.1555A>G and m.14846G>A with the opposite effect. Oxygen consumption rate associated with variants of mtDNA that lead to oxidative phosphorylation uncoupling was studied by means of treatment of cells with 4-(trifluoromethoxy)-phenylhydrazone carbonyl cyanide (FCCP) and oligomycin A. FCCP intercepts protons and transports them via the internal mitochondrial membrane, passing the proton channel of complex 5. Oligomycin inhibits the proton channel, therefore abolishing ATP synthase F1 capacity to synthesize ATP. Potassium cyanide (KCN) suppresses cytochrome c oxidase and, thus, decreases electron transport in the respiratory chain. Treatment of cells with KCN and succinate resulted in the loss of effect of the abovementioned mutations on oxygen consumption.

Several mtDNA mutations were found to correlate with pro-inflammatory activation of monocytes, including homoplasmic m.1811G>A (mitochondrial 16S rRNA gene) and m.9477G>A (cytochrome c oxidase gene) and heteroplasmic m.14459G>A (ND6 gene), m.1555A>G (mitochondrial 12S rRNA) and m.12315G>A [61]. Several of these mutations were able to change the activation of monocyte-derived macrophages via mitochondrial dysfunction in atherosclerosis. Mutations m.1555A>G and m.14846G>A were shown to be responsible for FCCP-induced increase in oxygen consumption.

The relationship between mitophagy abnormalities and mtDNA mutations has been studied by several research groups. Overall, mtDNA mutations compromising mitochondrial function were found to stimulate mitophagy, probably as compensatory mechanism to deactivate dysfunctional organelles [62]. Studies on cybrid lines identified a spectrum of mtDNA mutations associated with enhanced mitophagy initiation [63]. For the mutation m.13514A>G, it was shown that increased mitophagy was accompanied by inhibition of mitochondrial Ca^2+^ uptake and AMPK signaling, revealing more details on the mechanisms of increased mitophagy associated with mtDNA mutations [64]. Our group has conducted numerous experiments in cybrid lines focusing on atherosclerosis-related mtDNA mutations (detected in atherosclerotic patients). Several such mutations were shown to be associated with increased mitophagy, as assessed by LAMP expression: m.3336T>C, m.3256C>T, m.5178C>A, and m.5178C>A [14].

## 5. The Role of Mitochondria in Different Cell Types

### 5.1. Endothelial Cells

Endothelial cells (ECs) form the lining of blood vessels separating the vascular wall from circulating blood. The key functions of the ECs are to support vessel homeostasis, regulate vascular wall permeability and provide cellular transport [65]. Moreover, ECs take part in the inflammatory response in the arterial wall. Activation of the ECs in response to certain factors, such as signaling molecules, injury or changes of the laminar blood flow leads to increased production of cytokines and adhesion molecules [66]. Among the cytokines known to be produced by activated ECs are interleukin (IL)-3, IL-7, IL-8, IL-11, IL-14, IL-15, tumor necrosis factor (TNF)-α, transforming growth factor (TGF)-β, and granulocyte-macrophage colony stimulating factor (GM-CSF). Consequently, circulating immune cells can migrate to the site of cell activation and penetrate into the vessel wall [67]. There is a phenotypical and morphological variety of ECs within the arterial wall endothelium, which may also reflect deviations of the endothelial function [68]. In the ECs, only 2–6% of the cytoplasm volume is occupied by mitochondria, which appears to be low in comparison to other cell types with higher metabolic activity levels. Mitochondrial morphological changes in the ECs were shown to be associated with alteration of mitochondrial function and cellular metabolism. High expression of Drp1, affecting mitochondrial turnover, in vascular endothelium can induce the pro-inflammatory factors, and further damage the vessels. Endothelial dysfunction-related proteins are iNOS (inducible nitric oxide synthase), ICAM-1 (inter-cellular adhesion molecule 1) and vWF (von Willebrand factor). iNOS is one of the key enzymes generating nitric oxide (NO) from the amino acid l-arginine. ICAM-1 is a transmembrane protein, which is known to be up-regulated under inflammatory conditions in the ECs and epithelial cells. It mediates the adhesion of circulating immune cells and is considered as a marker of vascular EC activation or damage. Level of circulating vWF is increased following endothelial cell damage [69]. Hyperhomocysteinemia (HHcy) is a risk factor for peripheral vascular disorders [70]. Oxidative stress and activation of proinflammatory factors mediate the atherogenic effects of HHcy. It was shown that diabetes was associated with altered morphology of the mitochondria in the ECs, hepatocytes, and skeletal muscle, with increased mitochondrial fragmentation, reduced size and swelling [71,72].

ECs take part in oxygen transport from the blood to underlying tissues and are therefore highly exposed to oxygen. Relatively low consumption of oxygen by the EC mitochondria provides for effective oxy-gen transport from the blood vessel and protects the surrounding tissues from oxidative stress. Mechanisms of rapid oxygen transport through the vessel endothelium are currently being investigated [73].

Elevated levels of vascular endothelial growth factor (VEGF) were shown in hypoxia, which can promote the biogenesis of mitochondria. Moreover, this process includes protein kinase B (Akt) signaling. Akt is a key protein kinase that regulates cell metabolism and orchestrates the signaling cascades promoting cell survival, motility, and cell cycle progression. In turn, Akt kinase is downstream of the phosphoinositide (PI) 3-kinase signaling, which responds to insulin and other growth factors. Energy balance plays important regulatory function in angiogenesis driving blood vessel growth and branching. For instance, sirtuin 1 (Sirt1) is a highly conserved NAD+-dependent deacetylase, which is an important metabolic/energy sensor [74]. Suppression of *SIRT1* gene decreases biogenesis of mitochondria in the ECs, leading to reduction of vessel branching. Another important regulator of mitochondrial activity in the ECs is uncoupling protein 2 (UCP-2), which is a mitochondrial anion carrier protein. UCP-2 facilitates anion transfer from the inner mitochondrial membrane and proton transfer of opposite direction, therefore reducing the mitochondrial membrane potential [75].

### 5.2. Macrophages

Macrophages are key players of the innate immunity that respond to pathogen invasion and the appearance of dysfunctional and damaged cells by phagocytosis. Due to their phagocytic activity, macrophages actively participate in lipid accumulation in the arterial wall in atherosclerosis. Lipid uptake by macrophages goes via unspecific phagocytosis rather than specific receptor-mediated route, which leads to accumulation of undegraded cholesterol and lipids in the cytoplasm and foam cells formation. Pathogen detection and inflammatory response triggering is mediated by pattern-recognition receptors (PRRs) that are expressed by the immune cells [76]. These receptors identify pathogen-associated molecular patterns (PAMPs) that consist of components of the bacterial cell wall, nucleic acids, and proteins and induce classic activation of macrophages. However, PRRs also recognize the endogenous DAMPs that appear as a result of cellular damage, for example, free mtDNA, and urates [77].

Inside the cell, the effectors and adaptors of PRRs are situated close to the mitochondrial membrane. After the activation of TLRs, conserved signaling intermediate in Toll pathways (ECSIT) interacts with the tumor necrosis factor receptor (TNFR)-associated factor 6 (TRAF6). The adaptor protein TRAF6 is involved in many protein-protein interactions through its TRAF domain and a newly identified (RING) finger domain, which is a zinc finger-type domain with non-conventional E3 ubiquitin ligase activity [78]. ECSIT is also included in the mitochondrial respiratory complex I and contributes to mitochondrial ROS (mtROS) formation. Moreover, mtDNA promotes stimulation of NFκ-B and enables the expression of pro-inflammatory genes, which are responsible for the expression of such inflammatory mediations as TNF-α and IL-6. TNF-α is produced by macrophages in case of acute inflammation and participates in cellular signaling events, leading to necrosis or apoptosis. IL-6, in turn, has a pleiotropic effect on inflammation, immune response, and hematopoiesis [79].

Production of mtROS also promotes stimulation of NLRP3 inflammasomes, which, in turn, triggers sterile inflammation. By contrast, NFκ-B can activate mitophagy that can decrease NLRP3-triggered inflammatory responses in macrophages. Moreover, NLRP3 inflammasomes increase the level of PFKFB3 according to IL-1β-dependent pathway and modulate glycolysis [80].

Macrophage polarization in response to specific stimuli and signals is dependent on cellular metabolism and bioenergetic profile. Deregulation of this process contributes to different pathologies. Excessive pro-inflammatory activation of macrophages has damaging effects associated with uncontrolled inflammation. Persistence of pro-inflammatory activation leading to chronic inflammation is known to be associated with a number of chronic diseases, including rheumatoid arthritis, psoriasis, and inflammatory bowel disease. Mechanisms limiting excessive pro-inflammatory polarization of macrophages include signaling of anti-inflammatory cytokines, such as IL-10, therefore forming a balance between pro- and anti-inflammatory polarization (M1 and M2 respectively) [81].

Pro-inflammatory M1 macrophages produce large quantities of ROS, and this process is used not only to kill pathogens, but also to regulate cellular reactions. In M1 macrophages, iNOS contributes to NO production. M1 macrophages use mainly aerobic glycolysis for energy production, which is accompanied by a decrease of isocitrate dehydrogenase 1 (IDH1) and accumulation of succinate. Up-regulation of the pentose phosphate pathway (PPP) is one the reasons why M1 macrophages produce large quantities of ROS. PPP has a prominent antioxidative function through generation of NADPH. However, it also provides the precursors for nucleotide and amino acid synthesis necessary for growth [82]. By contrast, M2 macrophages contribute to tissue repair and homeostasis. They are characterized by high arginase activity and production of ornithine, which promotes the tissue repair, and use glycolysis to produce fatty acids [83]. Therefore, metabolism, which is dependent on correct mitochondrial functioning, has an important role in macrophage polarization. Immunometabolism became an attractive research topic and appears to be useful for developing new approaches for treatment of human inflammatory diseases. 

It should be noted that the bipolar classification of M1 and M2 types of macrophages is simplified and outdated. Besides M1 and M2 macrophages, other types exist that take part in pathogenesis of atherosclerosis. For example, Mox and Mhem macrophages differ in their response to oxidized phospholipids. The Mox cells regulate the redox status. Mhem macrophages, in turn, control iron metabolism in case of intraplaque hemorrhage. Moreover, macrophage populations can be different in humans and mice, further contributing to the variety [84].

### 5.3. Pericytes and Vascular Smooth Muscle Cells

The wall of human blood vessel contains three layers: tunica intima, tunica media, and tunica adventitia. The intimal layer, in turn, consists of the endothelial cell lining, subendothelial layer, and internal elastic lamina. The subendothelial layer of large blood vessels contains two main resident cell types: VSMCs (approximately 50–60% of total amount of cells) and macrovascular pericytes (up to 30% of cells). Both of these cell types express smooth muscle α-actin, which is a marker of contractile, non-proliferative phenotype [85]. However, functions of VSMCs and pericytes differ significantly. VSMCs are responsible for the coordination of physiologic processes required for homeostasis, for example, regulation of vascular tone and blood pressure. Pericytes support blood vessel integrity and normal functioning of the endothelial lining, forming multiple connections with the ECs [86,87]. These cells form a three-dimensional network being connected to each other and to the ECs and represent a second line of defense against pathogens under the endothelial lining [88]. The supportive function of pericytes is illustrated by their role in the survival of ECs of retinal capillaries. Pericyte loss caused by exposure to superoxide in diabetic retina, where it is most studied, is associated with microaneurysms and appearance of acellular capillaries [89]. Pericytes are characterized by contractile and synthetic activity but can also take part in phagocytosis and accumulation of LDL particles during atherosclerotic plaque development thus contributing to foam cell formation. Furthermore, pericytes, as dendritic cells, take part in antigen presentation and innate immune reactions [88].

Platelet-derived growth factor (PDGF) is an important regulatory protein involved in oxidative stress response. PDGF was shown to protect neurons from H_2_O_2_-induced oxidative stress through PI3K-Akt and MAP kinase signaling pathway [90]. In VSMCs, PDGF plays a key role in inducing phenotypic switching from contractile to proliferative state. The contractile phenotype has a low proliferation rate and reduced capacity for the formation of the extracellular matrix components, but produces contractile proteins smooth muscle myosin heavy chain, smooth muscle α-actin, and calponin. VSMCs of synthetic phenotype appear in case of vessel injury. They can proliferate and migrate towards the injury site. Moreover, they synthesize the extracellular matrix structural elements [91].

Studies on retinal microvascular pericytes have long demonstrated that oxLDL has toxic effects on these cells, which are associated with mitochondrial dysfunction [92]. More recent studies have revealed that the increased phagocytic activity of vascular wall cells induced by modified LDL also involves macrovascular pericytes that reside in the subendothelial layer [93]. In VSMCs, oxLDL can induce apoptosis, greatly contributing to the pro-inflammatory environment and the formation of unstable plaque [48]. Interestingly, stimulation of mitophagy was shown to have a protective effect in dysfunctional VSMCs, indicating that stimulation of this process may help reducing the risk of unstable plaque development [48,51].

## 6. The Mitochondrion as a Potential Therapeutic Target

The beneficial clinical effects of various drugs that are routinely used for treatment of diabetes and atherosclerosis appear to be conveyed, at least partially, by their anti-inflammatory effects. The study of pleiotropic effects and side effects of the common drugs is important for correcting and improving current therapeutic schemes. For instance, statins, which are widely used in patients with dyslipidemia, are also potent anti-inflammatory agents. However, statis were shown to interfere with mitochondrial pathways, inhibiting the respiratory chain and inducing mitochondrial apoptosis. The resulting increased oxidative stress and reduced energy production may explain some of the side effects of these drugs, as reviewed recently [94]. Together these observations indicate that the effect of statins may be biphasic, with positive and negative effects dependent on the dose—a phenomenon known as hormesis, which has been demonstrated for various substances and biologic processes. It is therefore important to develop treatments that will allow alleviating the toxic side effects of statins on the mitochondria. Recently, cell-permeable succinate was shown to restore mitochondrial respiration in experimental cellular models of statin toxicity [95]. These promising results may lead to improvement of statin therapy in the future. Metformin, a common anti-diabetic drug, is another example of medication with pleiotropic anti-inflammatory effects. Direct anti-inflammatory activity of metformin was demonstrated, and its beneficial effect on the mitochondrial metabolism in immune cells was described recently [96]. Statins, metformin, and other drugs used for treatment of cardiovascular diseases and diabetes, including polyphenols, were shown to modulate adenosine monophosphate-activated protein kinase (AMPK), which is a key cellular energy sensor [97]. That is not surprising, since AMPK is situated at the crossroads of multiple signaling pathways regulating cellular metabolism. However, it is important to consider the crosstalk between AMPK signaling and mitochondrial function, which may appear to be relevant for therapy improvement.

Currently, physical exercise is the most affordable and, at the same time, effective means of alleviating mitochondrial dysfunction in physically fit patients. Physical exercise has profound impact on cellular energy metabolism through AMPK signaling, which activates beta-oxidation of lipids, autophagy, and other processes to restore the energy homeostasis. The AMPK link was demonstrated between exercise and mitochondrial turnover with mitophagy activation, which helped refreshing the mitochondrial pool [98]. The effect of exercise, such as that of statins, can be considered as hormetic, since it causes transient elevation of ROS stimulating mitochondrial biogenesis and function (a process known as mitohormesis) but also improves mitophagy [99,100]. The increasing incidence of cardiovascular disorders can be attributed, at least partially, to the lack of exercise, which stimulates mitochondrial function, resulting in anti-inflammatory effect. In combination with excessive food consumption and obesity, which is associated with chronic inflammation, lack of mitochondrial stimulation through exercise can lead to deleterious effects. Together, these observations highlight the important role that regular physical exercise can play in the prevention and treatment of conditions associated with mitochondrial dysfunction and impaired mitochondrial turnover. This possibility is being actively explored and has already brought promising results for treatment of diabetes mellitus [101]. The beneficial effect of regular exercise (either alone or in combination with therapy) on cardiovascular risk, as well as on obesity and diabetes indices, was demonstrated for diabetes patients in a controlled trial [102]. It is also likely that regular exercise can potentiate the effect of commonly used medications. Development of treatment schemes taking these possibilities into account is an important future direction of atherosclerosis treatment.

Another important metabolic regulator is the group of peroxisome proliferator-activated receptors (PPARs), consisting of α, β, and γ isoforms. PPARs are transcription factors regulating multiple genes involved in glucose and lipoprotein metabolism and, correspondingly, mitochondrial function regulation. PPAR signaling is tightly linked to PGC-1α, which was identified as co-activator of PPARs in pathways increasing fatty acid oxidation. The PPARγ/PGC-1α signaling was shown to increase mitochondrial uncoupling and thermogenesis, being involved in the process of fat browning and response to exercise. PGC-1α also interacts with NRF1 and NRF2 stimulating mitochondrial biogenesis. These processes that appear to be important in the context of atherosclerosis development have been recently reviewed elsewhere [103]. The effects of PPAR agonists currently used in clinical practice for treatment of cardiovascular and metabolic diseases, such as fibrates (mostly activating PPARα) or glitazones (mostly activating PPARγ) should be considered [104].

Therapies directly targeting mitochondrial dysfunction are currently being actively developed. Such therapies appear to be promising for treatment of different chronic human diseases, including atherosclerosis [105]. The growing list of such therapies includes mitochondrial antioxidants and mitochondrial dynamic modifiers [106,107]. Antioxidant compounds are widely used for treatment of cardiovascular diseases, diabetes mellitus, and other disorders and have also been tested for atherosclerosis. However, most of these agents failed to demonstrate efficacy for atherosclerosis in clinical trials. Possible reasons for the observed weak effect of antioxidants could be explained by their indirect action on the mitochondrial oxidative stress and limited applicability depending on the patient’s redox status and mitochondrial function. That highlights the need for specific mitochondrial antioxidants available for clinical use. Coenzyme Q10 and its analogues were found to be promising to treat conditions associated with mitochondrial dysfunction [108,109]. However, more promising are specific mitochondrial antioxidants, such as mitoQ, which is able to associate with lipophilic molecules and, as a result, act on the mitochondria selectively. Other molecules of this group are superoxide dismutase mimetic agents EUK-8 and EUK-134 [110,111]. Furthermore, mitochondria-targeting quinones from SkQ family have been studied preclinically. These molecules have antioxidant properties reducing ROS formation and protecting cells from damage, as demonstrated in rat neurons [112].

Photo-sensibilization can be used as a tool to treat various pathological conditions. A relatively new technique called photo-theranostics can cause selective apoptotic cell death in cells and tissues that are exposed to pathological alterations. Selective accumulation of photo-sensitizing substance in cellular components allows to target them selectively at sub-cellular level. Molecules that can be used for photo-theranostics in case of mitochondria have already been discovered [113]. Protoporphyrin IX (PpIX) is a well-known photosensitizer [114]. This agent is generated in the mitochondria from a precursor, 5-aminolevulinic acid (5-ALA). Application of exogenous 5-ALA leads to PpIX enrichment which depends on the cellular metabolic profile and is already used for treatment of some cancers [115]. However, this approach is currently at the early development stage, and its clinical relevance is still unclear.

## 7. Conclusions

Mitochondrial dysfunction is currently recognized as an important therapeutic target for treatment of chronic human disorders, including atherosclerosis. Mitochondrial dysfunction can be caused by mtDNA mutation which can be inherited (as described for maternally inherited syndromes, such as MELAS and MIDD) or spontaneously acquired and accumulated during lifespan in different organs and tissues. The effects of mtDNA mutations can be more or less pronounced depending on the affected organ or tissue and also environmental factors, including the patient’s lifestyle. In the arterial wall, mtDNA mutations and associated mitochondrial dysfunction are involved in atherosclerosis development both at the initial stages, consistent of local activation and pro-inflammatory signaling, and at advanced stages, when massive lipid accumulation takes place. Moreover, mitochondrial dysfunction can contribute to unstable plaque development through maintaining chronic inflammatory conditions. Some of the identified mtDNA mutations associated with atherosclerosis have been already tested as potential disease markers, and more mtDNA mutations relevant for diagnostic purposes may appear in the future. Recent studies have tested the effects of commonly used drugs for improving the mitochondrial function. Moreover, mitochondria-targeting therapies, such as selective antioxidant therapies, are underway for clinical testing. Evaluation of therapeutic effects of healthy lifestyle and exercise to correct mitochondrial dysfunction is another promising future direction.

## Data Availability

Not applicable.
